# 

**Published:** 1838

**Authors:** 


### MEDICAL PROFESSORS

IN THE

### CINCINNATI COLLEGE,

AS

EDITORS, PROPRIETORS AND PUBLISHERS,

OF THE

### WESTERN JOURNAL:

DANIEL DRAKE, M. D.
JOHN P. HARRISON, M. D.
JAMES B. ROGERS, M. D.
LANDON C. RIVES, M. D.
WILLARD PARKER, M. D.
JOSEPH N. McDOWELL, M. D.
SAMUEL D. GROSS, M. D.

---

EDITORIAL COMMITTEE FOR THE PRESENT VOLUME:

DRS. DRAKE, HARRISON AND GROSS.

---

PUBLISHING COMMITTEE:

DRS. DRAKE AND RIVES.

---

### TERMS OF PUBLICATION.

All letters of business must be directed (post paid) to the last named Committee. Subscription price $3 payable on the receipt of the first number of the volume. The notes of all solvent banks received at par, if remitted free of postage.

### TRAVELLING AGENTS FOR THE WESTERN JOURNAL.

C. W. James—the Eastons, brothers—C. Tobey: all of this city.

---

### TERMS.

Published quarterly, in numbers of 160 pages, price three dollars payable on the delivery of the first number.

